# Recombinant Acetylcholine Receptor Immunization Induces a Robust Model of Experimental Autoimmune Myasthenia Gravis in Mice

**DOI:** 10.3390/cells13060508

**Published:** 2024-03-14

**Authors:** Lukas Theissen, Christina B. Schroeter, Niklas Huntemann, Saskia Räuber, Vera Dobelmann, Derya Cengiz, Alexander Herrmann, Kathrin Koch-Hölsken, Norbert Gerdes, Hao Hu, Philipp Mourikis, Amin Polzin, Malte Kelm, Hans-Peter Hartung, Sven G. Meuth, Christopher Nelke, Tobias Ruck

**Affiliations:** 1 Department of Neurology, Medical Faculty and University Hospital Duesseldorf, Heinrich-Heine University Duesseldorf, Moorenstr. 5, 40225 Duesseldorf, Germany; lukas.theissen@med.uni-duesseldorf.de (L.T.); christinabarbara.menskes@med.uni-duesseldorf.de (C.B.S.); niklas.huntemann@med.uni-duesseldorf.de (N.H.); saskiajanina.raeuber@med.uni-duesseldorf.de (S.R.); vera.dobelmann@med.uni-duesseldorf.de (V.D.); derya.cengiz@med.uni-duesseldorf.de (D.C.); alexander.herrmann@med.uni-duesseldorf.de (A.H.); kathrin.koch@med.uni-duesseldorf.de (K.K.-H.); hans-peter.hartung@uni-duesseldorf.de (H.-P.H.); svenguenther.meuth@med.uni-duesseldorf.de (S.G.M.); 2 Department of Cardiology, Pulmonolgy and Vascular Medicine, Medical Faculty and University Hospital Duesseldorf, Heinrich-Heine University Duesseldorf, Moorenstr. 5, 40225 Duesseldorf, Germany; gerdes@hhu.de (N.G.); hao.hu@med.uni-duesseldorf.de (H.H.); philipp.mourikis@med.uni-duesseldorf.de (P.M.); amin.polzin@med.uni-duesseldorf.de (A.P.); malte.kelm@med.uni-duesseldorf.de (M.K.); 3 Brain and Mind Center, University of Sidney, Sidney NSW 2050, Australia; 4 Department of Neurology, Palacky University Olomouc, 77146 Olomouc, Czech Republic

**Keywords:** myasthenia gravis, complement, murine model, experimental autoimmune myasthenia gravis

## Abstract

Myasthenia gravis (MG) is a prototypical autoimmune disease of the neuromuscular junction (NMJ). The study of the underlying pathophysiology has provided novel insights into the interplay of autoantibodies and complement-mediated tissue damage. Experimental autoimmune myasthenia gravis (EAMG) emerged as a valuable animal model, designed to gain further insight and to test novel therapeutic approaches for MG. However, the availability of native acetylcholine receptor (AChR) protein is limited favouring the use of recombinant proteins. To provide a simplified platform for the study of MG, we established a model of EAMG using a recombinant protein containing the immunogenic sequence of AChR in mice. This model recapitulates key features of EAMG, including fatigable muscle weakness, the presence of anti-AChR-antibodies, and engagement of the NMJ by complement and a reduced NMJ density. Further characterization of this model demonstrated a prominent B cell immunopathology supported by T follicular helper cells. Taken together, the herein-presented EAMG model may be a valuable tool for the study of MG pathophysiology and the pre-clinical testing of therapeutic applications.

## 1. Introduction

Myasthenia gravis (MG) is an autoimmune disease affecting the transmission at the neuromuscular junction (NMJ). The pathophysiology of MG is characterized by pathogenic antibodies targeting proteins of the postsynaptic membrane [1], with 80 to 90% of cases mediated by antibodies directed against the acetylcholine receptor (AChR) [2].

MG emerged as a prototype disease for the study of antibody-mediated complement activation. The knowledge of specific disease mechanisms allowed for the development of tailored treatment approaches, such as complement blockade [3] or inhibition of the neonatal Fc receptor [4]. The clinical efficacy of these approaches underlines the critical value of in-depth knowledge on disease pathophysiology.

In part, evidence for complement activation playing a critical role in amplifying the immune response and exacerbating the disease originated from murine models of MG [5,6]. Among these, experimental autoimmune myasthenia gravis (EAMG) is a well-established preclinical model used to study the pathogenesis of MG and test therapeutic interventions. A potential drawback to this model is the limited availability of AChR needed for immunization. The majority of current EAMG models employ AChR obtained from electric eels, such as *Torpedo californica* (also called *Tetronarce californica*) [7]. The handling of these animals and the isolation of viable AChR is challenging, prompting the need for alternative strategies. One approach is the use of recombinant AChR [8,9]. However, only a few studies characterized the use of recombinant AChR and, to the best of our knowledge, none employed mice as model organisms [7].

The EAMG model is a valuable platform to study the prototypical interplay of pathogenic antibodies and complement-mediated tissue damage, with broader implications for other autoimmune disorders with shared mechanisms. To improve the availability and reproducibility of this model, we aimed to create an EAMG protocol that (1) employs recombinant AChR as antigen, (2) utilizes mice as readily available laboratory animal with the opportunity to use a plethora of transgenic mouse strains, (3) produces a clinical phenotype allowing the quantification of disease impairment, and (4) demonstrates antibody-engagement and complement-mediated damage at the NMJ.

## 2. Methods

### 2.1. Experimental Autoimmune Myasthenia Gravis (EAMG)

All animal experiments were approved by the local animal welfare officers (81-02.04.2021.A408). For this project proposal, we constructed an immunization model of EAMG [7,10]. C57BL/6 mice were commercially acquired and immunized with 40 µg of the acetylcholine receptor subunit alpha (CHRNA1) (Cusabio, Houston, TX, USA) emulsified in 100 µL of phosphate-buffered saline (PBS) and 100 µL of complete Freund’s adjuvant (CFA) (Sigma-Aldrich Co., St. Louis, MO, USA). The latter was prepared by adding 100 µg Mycobacterium tuberculosis to 100 µL complete Freund’s adjuvant. We injected 50 µL of solution subcutaneously on both flanks and 100 µL at the base of the tail. Mice were immunized twice: at 8 weeks of age and at 12 weeks of age. At both time points, we employed CHRNA1 together with CFA, as described above. For control, C57BL/6 mice were immunized following the same protocol using only CFA and PBS, but no CHRNA1 antigen. We employed 12 mice per group. Mice were scored biweekly by measuring the forelimb grip strength before and after exertion with a Grip Strength Meter. Here, we determined grip strength based on a total of 6 measurements: three taken before and three taken after exhaustion. Mice were secured by their tail, allowing them to grasp the grid only with their forepaws. Subsequently, the mouse was pulled horizontally from the grid with consistent force. As readout, we calculated the mean grip strength of the three values obtained after exhaustion. Exhaustion was created by retracting mice 20 times from their cages after allowing them to hold to the metal bars with the forelimbs. The raters were blinded for the group assignment of the mice with the mice only labelled by a number. Mice used for the experiments were housed in individually ventilated cages with EAMG mice and controls mixed together, to not allow for identification by the raters. Six mice were housed in each cage. During regular twelve hour light/dark cycles, mice were supplied with water and food ad libitum. Mice were checked daily. Mice were additionally rated according to the EAMG score (0: No clinical change, 1: Mild muscle weakness after exercise, 2: Muscle weakness before exercise, 3: Severe muscle weakness with dyspnea/apnea). At 18 weeks of age, mice were sacrificed and blood as well as skeletal muscles were harvested for further analysis.

### 2.2. Immunofluorescence

Immunofluorescence (IF) was performed as previously described [11]. Briefly, all stainings were performed on 8 µm cryostat sections. We used irrelevant antibody stains (either mouse/rabbit monoclonal/polyclonal isotype controls) as negative controls, as well as omission of the primary antibody. The following antibodies were used for staining procedures: C3 (Novus biologicals), IgG (Novus biologicals) and α-bungarotoxin (Invitrogen). Specimens were analysed in cooperation with the Core Facility for Advanced Light Microscopy, Heinrich-Heine University Düsseldorf. For quantification of NMJs, we employed staining for α-bungarotoxin and neurofilament light chain (NFL, Novus biologicals). The biopsies were blinded for quantification with the group not possible to identify from the label. The scoring was performed in randomly distributed 10 high-power fields (HPF, based on the microscope used and the respective oculars ≙ 0.16 mm^2^), as previously described [12].

### 2.3. Flow Cytometry

The spleen, thymus and lymph nodes were collected and transferred into a 40 µm pore size cell filter (Greiner Bio-One, Kremsmünster, Austria) and then mechanically crushed with the plunger of a sterile 2 mL-syringe (B. Braun SE, Melsungen, Germany). The filter was rinsed with 10 mL wash medium (DMEM, 1% FCS, 1% penicillin/streptomycin) and the tubes were centrifuged at 1500 rpm for 5 min. For thymocytes and splenocytes, cells were treated with 5 mL ammonium–chloride–potassium (ACK) buffer for 60 s. The cells were washed in flow cytometry running buffer. The supernatant was discarded again, the cell pellet resuspended in 1 mL running buffer and filtered again through a 40 µm cell filter. Finally, the filter was rinsed and the cell count was determined using a cell counter (Bio-Rad, Hercules, CA, USA). For antibody staining, cells were then incubated with fluorochrome-conjugated antibodies at 4 °C for 30 min in the dark (Table 1). For intracellular staining, cells were treated with fixation/permeabilization solution (Thermo Fisher Scientific, Waltham, MA, USA) for 20 min, washed with permeabilization buffer (Thermo Fisher Scientific) and incubated with antibodies directed against intracellular target molecules of interest. For cytokine analysis, cells were cultivated overnight in X-VIVO™ 15 Serum-free Hematopoietic Cell Medium (Lonza, Basel, Switzerland). Then, cells were stimulated with Leucocyte Activation Cocktail, with BD GolgiPlug™ with added phorbol myristate acetate, ionomycin and brefeldin A (BD Biosciences, Heidelberg, Germany) for 4 h before staining. Cells were stained with extracellular lineage markers for identification and intracellular staining for the corresponding cytokine. Approximately 1 × 10^6^ freshly isolated cells were used per sample and staining. Cells were additionally stained with the Zombie NIR dye (Biolegend, San Diego, CA, USA) according to the manufacturer’s manual to distinguish between dead and live cells. All cells were washed and resuspended and analysed by flow cytometry using a CytoFlex Flow Cytometer (Beckman Coulter, Brea, CA, USA). Flow cytometric data was processed using Omiq^®^ (Version 1.1, Dotmatics, Boston, MA, USA).

### 2.4. Enzyme-Linked Immunosorbent Assay

Enzyme-linked immunosorbent assays (ELISA) were performed for the detection and quantification of anti-AChR-antibodies at 18 weeks of age. We used a commercially available ELISA according to the manufacturer’s instructions (Hölzel-Biotech, BM-EKC36236, Cologne, Germany).

### 2.5. Statistical Analysis

Statistical analysis was performed using GraphPad Prism 9.3 (GraphPad Software, Inc., San Diego, CA, USA). Group differences were assessed by Student’s *t*-test (for two categories) or analysis of variance (ANOVA) test (for more than two categories) as appropriate. Differences were considered statistically significant with the following *p*-values: * *p* ≤ 0.05, ** *p* < 0.01 and *** *p* < 0.001.

## 3. Results

### 3.1. CHRNA1 Immunization Induces A Murine Model of EAMG

**Figure 1:**
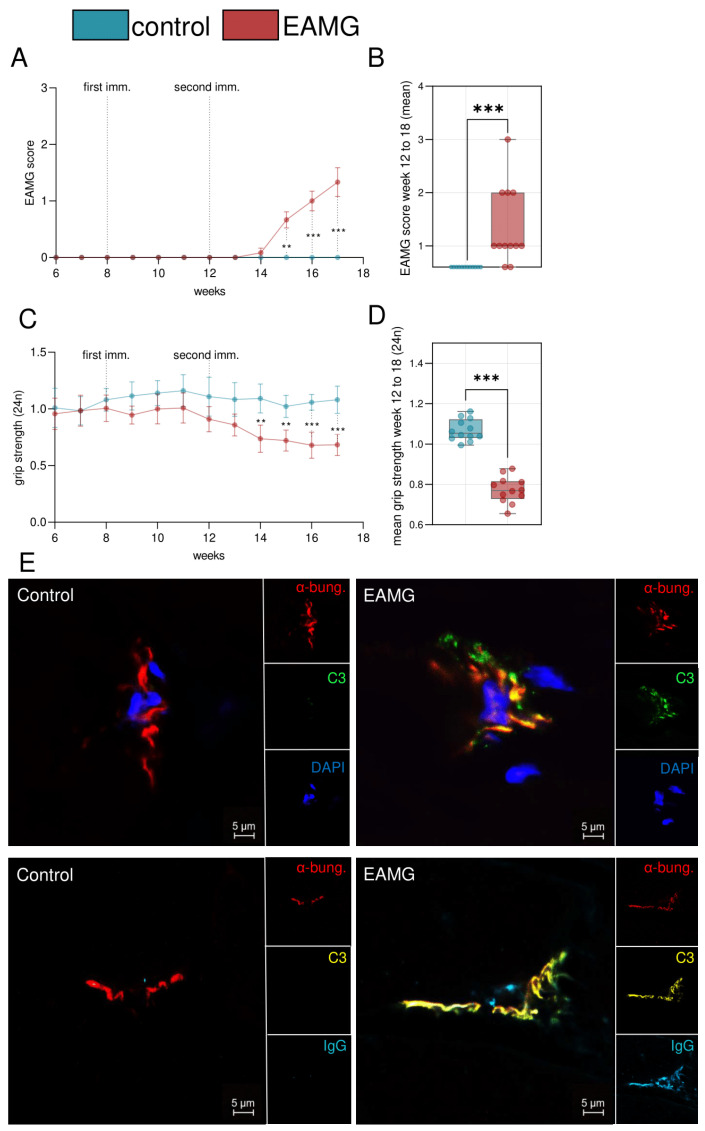
**A model of experimental autoimmune myasthenia gravis.** (**A**): Experimental autoimmune myasthenia gravis (EAMG) score scored weekly. Mice were immunized in week 8 and 12 with n = 12 per group. EAMG mice were immunized with CHRNA1 antigen and complete Freund’s adjuvant. Controls received only complete Freund’s adjuvant. (**B**): Mean EAMG score of weeks 12 to 18 for each group. (**C**): Forelimb grip strength was scored biweekly after exercising the animals. (**D**): Mean forelimb grip strength of weeks 12 to 18 for each group. Antibodies were quantified by enzyme-linked immunosorbent assay. (**E**): Immunofluorescence staining of representative muscle specimen of EAMG and control mice. α-bungarotoxin (α-bung) binds the acetylcholine receptor (red). Complement deposits are stained with C3 (green or yellow) and IgG (blue). Groups were compared by unpaired student’s *t*-test., *p* < 0.01 **, *p* < 0.001 ***, *p* > 0.05 not significant.

In order to establish a model for EAMG, we immunized C57BL/6 mice with recombinant CHRNA1 and CFA at eight weeks of age. CHRNA1 contains the immunogenic sequence of AChR [13]. Control mice were immunized with CFA, but not CHRNA1. We assessed the EAMG score, as previously described [7], following the first immunization at eight weeks (Figure 1A). Coinciding with the second immunization at 12 weeks, EAMG mice demonstrated a clinical phenotype as evidenced by an EAMG score of at least one. The EAMG score increased progressively. At 18 weeks, mice were sacrificed for further analysis. In contrast to EAMG mice, controls did not display a change to the EAMG score (Figure 1B). Out of 12 mice that were immunized with CHRNA1, 10 mice (83.3%) demonstrated an EAMG score of at least one.

**Figure 2:**
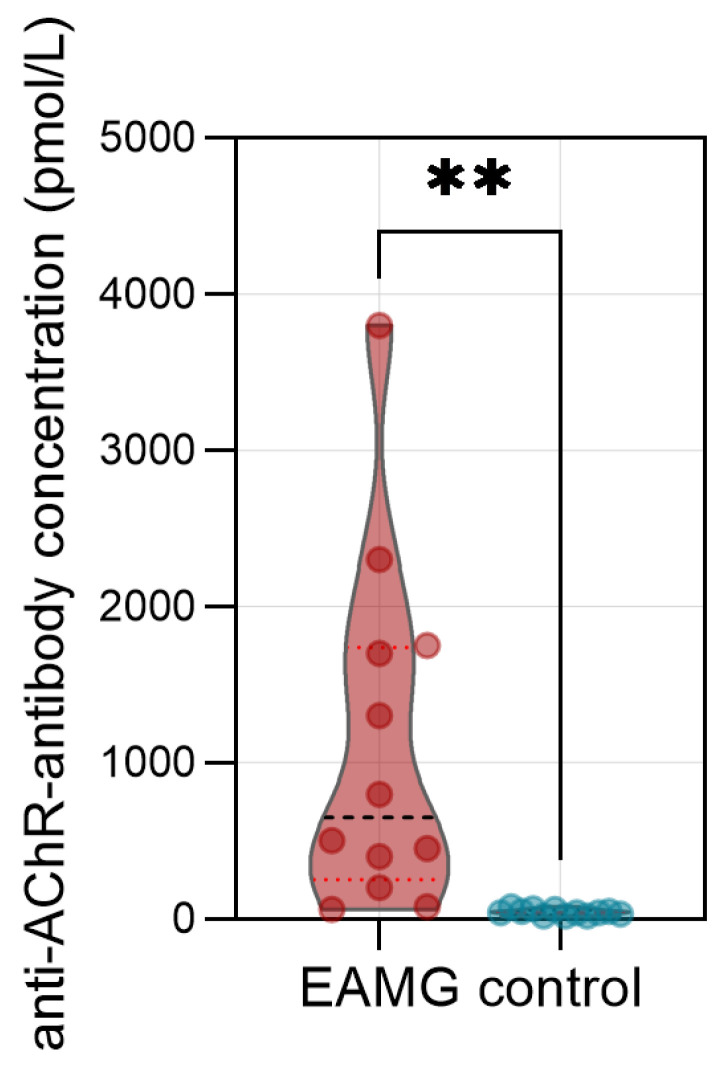
**Antibody levels in experimental autoimmune myasthenia gravis mice.** Levels of anti-acetylcholine receptor-antibodies in the serum of experimental autoimmune myasthenia gravis (EAMG) mice and controls. N = 12 per group. Antibodies were quantified by enzyme-linked immunosorbent assay. Groups were compared by unpaired student’s *t*-test-*p* ** < 0.01.

As additional clinical readout, we measured the forelimb grip-strength before and after exertion (Figure 1C). Following the second immunization at 14 weeks, EAMG mice displayed a reduced forelimb grip strength after exertion compared to control mice (Figure 1D). To assess antibody engagement at the post-synaptic membrane, we performed IF analysis of skeletal muscle using the quadriceps muscle of EAMG mice and controls (Figure 1E). The NMJ was localized by staining with α-bungarotoxin binding the AChR with high affinity. To demonstrate the formation of complement, we stained for complement protein C3. In all EAMG mice (n = 12), but in none of the controls (n = 12), we detected clear co-localisation of C3 with the NMJ. To corroborate the presence of pathogenic antibodies against the AChR, we also demonstrated the presence of IgG at the NMJ in EAMG mice, but not in controls. Further, we used an ELISA for quantification of anti-AChR-antibodies in the serum of experimental mice at 18 weeks of age (Figure 2). Here, anti-AChR-antibodies were detected in EAMG mice, but not in controls. Antibody levels were heterogeneous and ranged between 60 to 3740 pmol/L in EAMG mice and between 20 to 80 pmol/L in controls.

**Figure 3:**
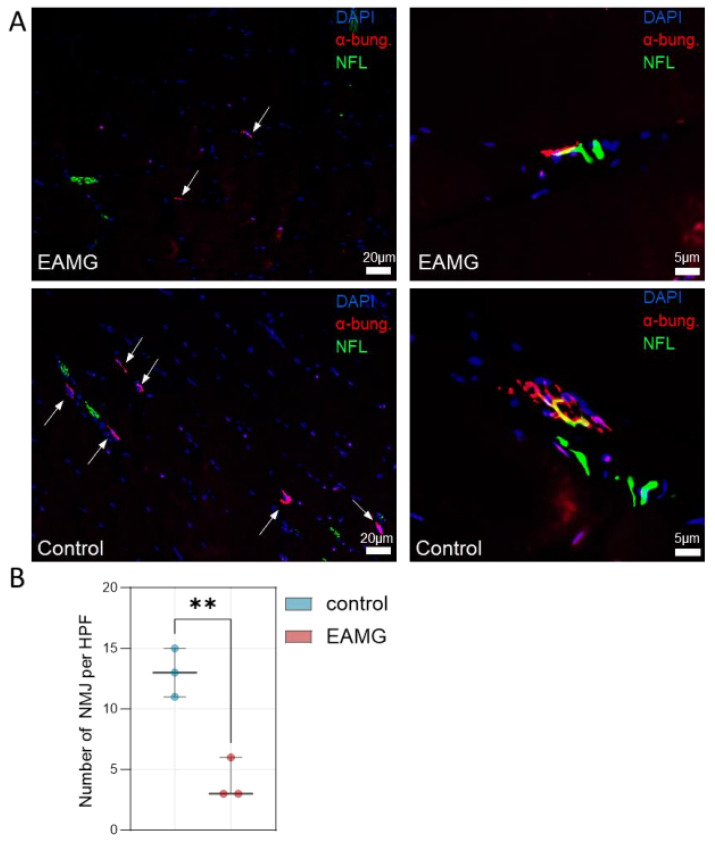
**EAMG mice demonstrate a reduced number of NMJs.** (**A**): Left: Immunofluorescence staining of representative muscle specimen of EAMG and control mice. α-bungarotoxin (α-bun) binds the acetylcholine receptor (red) while neurofilament light chain (NFL) binds to peripheral nerves (green). Right: Zoom in on exemplary NMJs highlighting a loss of topographic complexity in EAMG mice. (**B**): NMJs were counted in randomly distributed 10 HPF (≙0.16 mm^2^). The biopsies were blinded for quantification, with the group impossible to identify from the label. Groups were compared by unpaired Student’s *t*-test. *p* ≤ *p* < 0.01 **. Abbreviations: EAMG, experimental autoimmune myasthenia gravis; HPF, high-power field.

Finally, we also quantified the density of NMJs in EAMG and control muscle specimen (Figure 3A). EAMG mice demonstrated a reduced number of NMJ per HPF, while also showcasing a loss of NMJ complexity (Figure 3A,B). In EAMG, NMJ mostly appeared with a simplified topography and loss of their fold-like structure, as compared to control mice featuring complex NMJs with a “pretzel”-like appearance, as previously described [14].

Taken together, immunization with recombinant CHRNA1 appears a viable strategy to induce key features of EAMG in mice, e.g., a clinical phenotype of fatigable muscle weakness, complement engagement of the NMJ and an autoantibody response against AChR.

### 3.2. Activated B Cells Reside in Immunological Organs of EAMG

**Figure 4:**
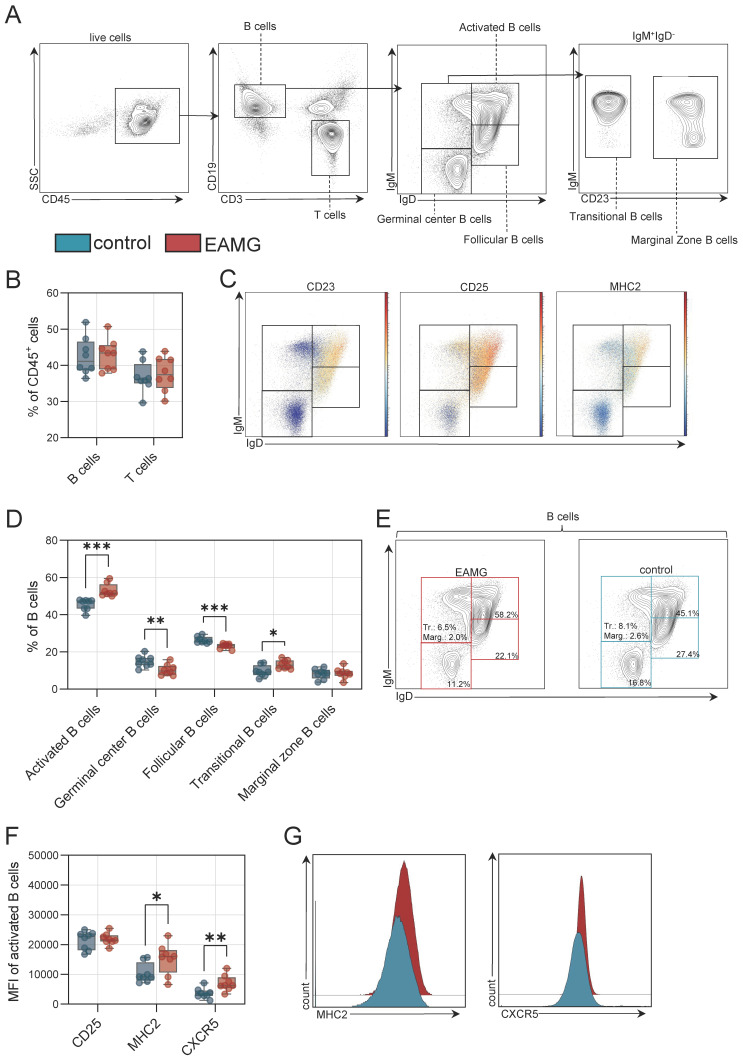

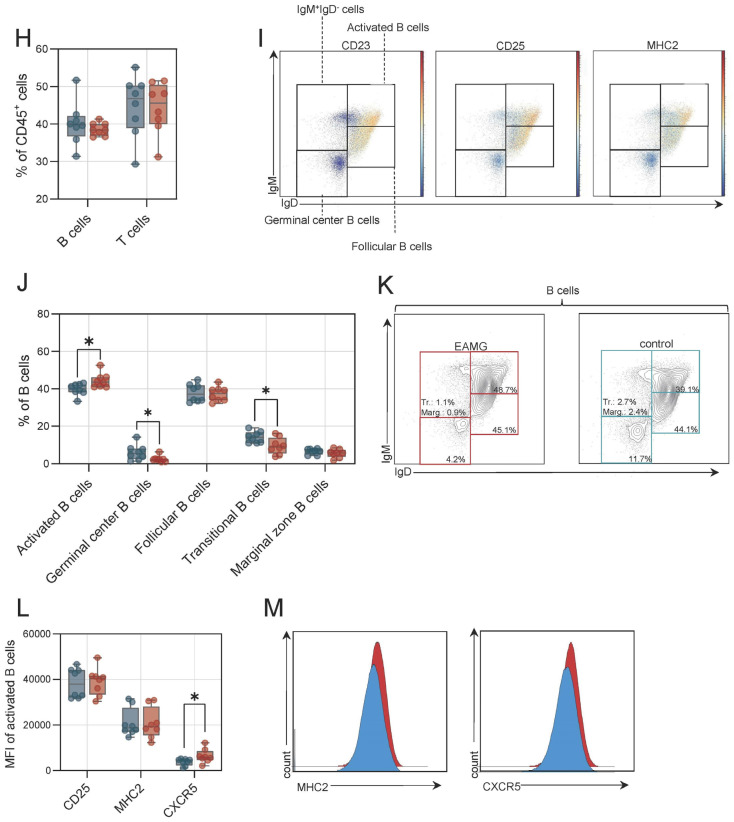
**B cell pathology in experimental autoimmune myasthenia gravis in the spleen and in draining lymph nodes.** (**A**): Representative gating for B cells and B cell subgroups. Live cells were identified by live/dead marker. (**B**): Frequency of B and T cells in the spleen of experimental autoimmune myasthenia gravis (EAMG) mice and controls. N = 8 per group. (**C**): Representative gating for CD23, CD25 and major histocompatibility complex (MHC2). Red indicates high expression; blue indicates low expression for each cell. (**D**): Frequencies of B cell subgroups in the spleen. (**E**): Representative gating for B cells of EAMG mice and controls. (**F**): Median fluorescence intensity (MFI) of CD25, MHC2 and CXCR5 (CD185). (**G**): Representative histograms for EAMG mice (red) and controls (blue) for MHC2 and CXCR5. (**H**): Frequency of B and T cells in the abdominal lymph nodes of experimental autoimmune myasthenia gravis (EAMG) mice and controls. N = 8 per group. (**I**): Representative gating for CD23, CD25 and major histocompatibility complex (MHC2). Red indicates high expression; blue indicates low expression for each cell. (**J**): Frequencies of B cell subgroups in lymph nodes. (**K**): Representative gating for B cells of EAMG mice and controls. (**L**): Median fluorescence intensity (MFI) of CD25, MHC2 and CXCR5 (CD185). (**M**): Representative histograms for EAMG mice (red) and controls (blue) for MHC2 and CXCR5. Groups were compared by unpaired Student’s *t*-test. *p* ≤ 0.05 *, *p* < 0.01 **, *p* < 0.001 ***, *p* > 0.05 not significant.

Next, we aimed to characterize the immunopathology in our EAMG model. For this purpose, we collected the spleen and abdominal LNs of EAMG mice and controls (n = 12 per group). After dissociation, cells were analysed for the B cell compartment (Figure 4A). Briefly, B and T cell populations were differentiated based on CD19 and CD3 levels. In spleen, we detected no differences in frequencies of B or T cells (Figure 4B). Next, we analysed canonical subtypes of B cells including activated (IgM^high^, IgD^high^, CD25^high^, major histocompatibility class II (MHC2)^high^), follicular(IgM^low^, IgD^high^, CD23^+^), germinal centre (IgM^−^, IgD^−^), transitional (IgM^high^, IgD^low^, CD23^−^) and marginal zone B cells (IgM^high^, IgD^low^, CD23^+^) (Figure 4A,C). In the spleen, B cell populations were shifted towards activated and transitional B cells in EAMG, while germinal centre and follicular B cell frequencies were decreased (Figure 4D,E). To further characterize the activated B cell phenotype in EAMG, we assessed surface levels of CD25, MHC2 and the CXC motif chemokine receptor 5 (CXCR5) in these cells (Figure 4F). Activated B cells had higher MHC2 and CXCR5 levels in EAMG compared to controls (Figure 4G). Moreover, we also sought to characterize the B cell compartment in EAMG in draining LNs as additional immune organ. Here, we observed no differences for B or T cell frequencies comparing EAMG and controls (Figure 4H). We applied the same flow cytometry panel to the B cell compartment as in the spleen (Figure 4I) and observed that EAMG is also characterized by a shift towards activated B cells in the abdominal LNs (Figure 4J,K). Conversely, germinal and transitional B cell frequencies were higher in controls compared to EAMG in LNs. Here, activated B cells also demonstrated higher levels of CXCR5 in EAMG (Figure 4L,M). Succinctly, the EAMG model demonstrates a B cell immunopathology characterised by a shift towards activated B cells expressing high levels of CXCR5.

### 3.3. Higher Plasma Cell Frequencies Are Detected in EAMG Spleen

**Figure 5:**
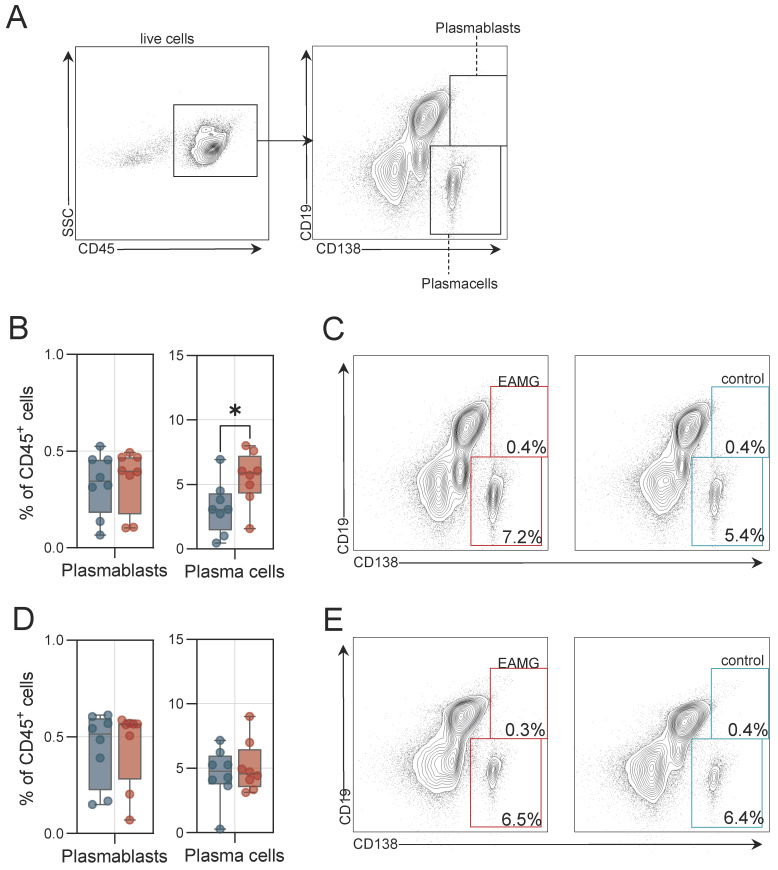
**Plasma cell expansion in the experimental autoimmune myasthenia gravis spleen.** (**A**): Representative gating of plasma cells and plasmablasts. Live cells were identified by live/dead staining. (**B**): Frequencies of plasma cells and plasmablasts of experimental autoimmune myasthenia gravis (EAMG) mice and controls in the spleen. N = 8 per group. (**C**): Representative gating for plasma cells in EAMG mice and controls in the spleen. (**D**): Frequencies of plasma cells and plasmablasts in the abdominal lymph nodes. (**E**): Representative gating for plasma cells in EAMG mice and controls in the abdominal lymph nodes. Groups were compared by unpaired student’s *t*-test. *p* ≤ 0.05 *, *p* > 0.05 not significant.

B cell differentiation results in the formation of plasma cells capable of secretion of pathogenic autoantibodies. We also sought to study these cells in immunological organs of EAMG. For this purpose, we analysed the spleen and abdominal LNs by flow cytometry. Plasma cells were defined as CD19^−^CD138^+^, while plasmablasts were defined as CD19^+^CD138^+^ (Figure 5A). In the spleen, frequencies of plasmablasts were similar between EAMG and controls, while plasma cells were increased in EAMG (Figure 5B,C). In abdominal LNs, we detected no differences between EAMG and controls in respect to plasmablast and plasma cell frequencies (Figure 5D,E). Together, EAMG mice display higher plasma cell frequencies in the spleen, but not in abdominal LNs.

### 3.4. The T Follicular Helper T Cell Compartment Is Altered in EAMG

**Figure 6:**
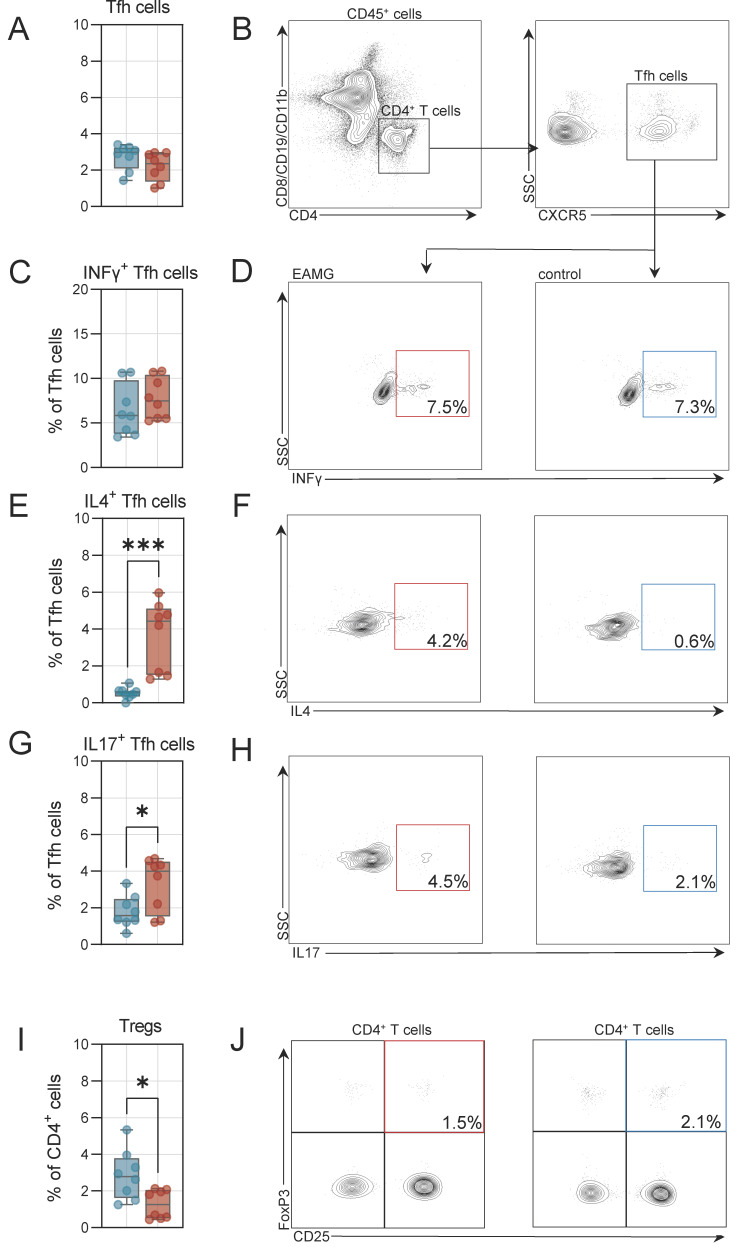

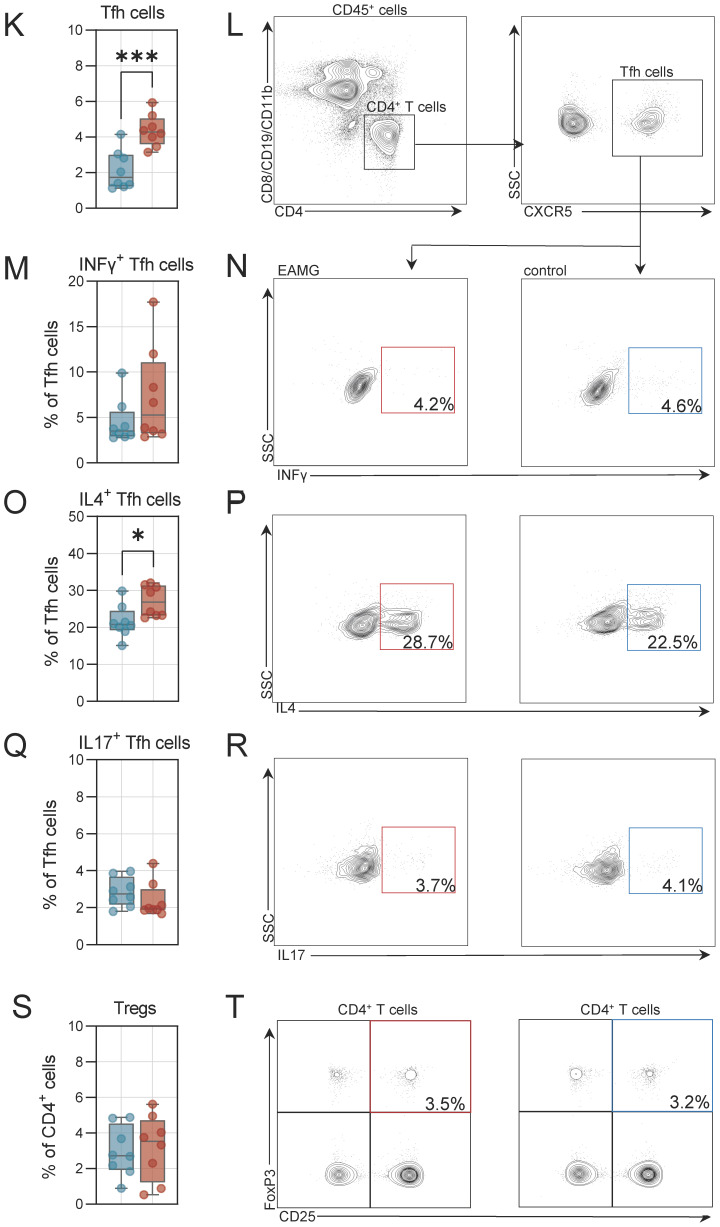
**Altered T helper cell properties in the spleen and in the draining lymph nodes.** (**A**): Frequencies of T follicular helper (Tfh) cells in experimental autoimmune myasthenia gravis (EAMG) mice and controls in the spleen. N = 8 per group. (**B**): Representative gating of Tfh cells. Live cells were identified by live/dead staining. (**C**,**D**): Frequencies of Interferon-γ (INFγ) positive Tfh cells and representative gating. (**E**,**F**): Frequencies of interleukin 4 (IL4) positive Tfh cells and representative gating. (**G**,**H**): Frequencies of interleukin 17 (IL17) positive Tfh cells and representative gating. (**I**,**J**): Frequencies of regulatory CD4 T cells (Tregs) and representative gating. (**K**): Frequencies of T follicular helper (Tfh) cells in experimental autoimmune myasthenia gravis (EAMG) mice and controls in the abdominal lymph nodes. N = 8 per group. (**L**): Representative gating of Tfh cells. Live cells were identified by live/dead staining. (**M**,**N**): Frequencies of Interferon-γ (INFγ) positive Tfh cells and representative gating. (**O**,**P**): Frequencies of interleukin 4 (IL4) positive Tfh cells and representative gating. (**Q**,**R**): Frequencies of interleukin 17 (IL17) positive Tfh cells and representative gating. (**S**,**T**): Frequencies of regulatory CD4 T cells (Tregs) and representative gating. Groups were compared by unpaired Student’s *t*-test. *p* ≤ 0.05 *, *p* < 0.001 ***, *p* > 0.05 not significant.

Finally, we also aimed to study the T follicular helper (Tfh) cell population. Tfhs are a specialized subset of antigen-experienced CD4 T cells critically involved in mediating the differentiation of B cells into memory B cells and plasma cells [15]. We determined the number of Tfh cells (defined as CD4^+^CXCR5^+^ and negative for CD8, CD19, CD11b dump channel) in the spleen and abdominal LNs. In the spleen, Tfh frequencies were similar for EAMG and controls (Figure 6A,B). Next, we also quantified the number of Tfh cells expressing intracellular signalling molecules required for B cell differentiation. Frequencies of interferon-γ (INFγ)^+^ Tfh cells were unchanged between EAMG and controls (Figure 6C,D). In contrast, the number of interleukin 4 (IL4)^+^ and interleukin 17 (IL17)^+^ Tfh cells were substantially higher in the spleen of EAMG mice as compared to controls (Figure 6E–H). Tfh cells are the major source of IL4 and IL17 and both cytokines are required for B cell differentiation and mediate somatic recombination [15,16]. Besides Tfh cells, we also assessed the number of regulatory T cells (Tregs), as these cells may dampen autoimmunity and enhance self-tolerance. These cells were defined as CD4^+^FoxP3^+^CD25^+^. Indeed, we recorded lower numbers of Tregs in the spleen of EAMG mice as compared to controls (Figure 6I,J). Further, we also analysed these cell populations in abdominal LNs. Here, Tfh cells were more frequent in EAMG mice compared to controls (Figure 6K,L). Analysis of intracellular cytokines indicated that IL4+ Tfh cells were also more abundant in LNs of EAMG, while INFγ^+^ and IL17^+^ Tfh cell numbers were unchanged (Figure 6M–R). In respect to Tregs, frequencies were comparable between EAMG and controls in abdominal LNs (Figure 6S,T). Taken together, the Tfh compartment is shifted towards the expression of IL4 in immunological organs of EAMG mice potentially driving the differentiation of B cells into plasma cells capable of autoantibody secretion.

## 4. Discussion

MG emerged as a prime example for the interplay of pathogenic autoantibodies and complement-mediated tissue damage. The knowledge of participating immune features allowed for the development of specific treatment strategies [3,4,17]. Extending this knowledge to other entities with shared disease-mechanisms may improve the management of the latter. However, before advancing to clinical trials, preclinical animal studies remain helpful to understand advantageous and disadvantageous strategies and test new treatment approaches.

The EAMG model demonstrates key mechanisms involved in the immune response against the NMJ including T cell dysfunction, B cell differentiation into plasma cells, antibody generation and complement-mediated tissue damage [18]. This renders the EAMG model a valuable tool for the study of specific pathomechanisms, as well as testing novel therapeutic approaches for MG.

A broad range of different models have been suggested, including the use of susceptible strains of rats or mice as well as the use of AChR isolated from electric eels, mammalian muscle or recombinant AChR fragments [7]. Among these models, the majority of studies made use of rats immunized with AChR recovered from electric eels [7,19]. While the use of rats compared to mice has inherent advantages and disadvantages, one asset that might favour the use of mice as experimental animals is the availability of tools to genetically manipulate their genomes [20]. As such, a murine model of EAMG that employs recombinant AChR might be a valuable platform both in terms of accessibility as well as genomic modularity. Following this line of argumentation, we report a model that covers key features of EAMG employing a murine background. Our model is based on the widely available C57BL/6 strain enabling genetic and pharmacological investigations of mechanisms of interest.

We also report that this model features a dysregulated T helper compartment as well as B cell differentiation and activation, in line with current knowledge on MG pathophysiology [21]. Here, we observed subtle differences in immune cell composition between the spleen and the lymph nodes. Specifically, in the spleen of EAMG mice, B cells shifted towards an activated phenotype with increased levels of MHC2 and CXCR5. In lymph nodes, this effect was less pronounced, with B cells also shifting towards activation and increased CXCR5 levels, but without changes to MHC2. Plasma cells were expanded in the spleen of EAMG mice, but not in lymph nodes. Concurrently, IL4^+^ Tfh cells were expanded in EAMG spleen and lymph nodes, while IL17^+^ cells were only increased in the spleen. Given the link between pathogenic antibodies and MG, B cells and their differentiation states are directly implicated in the disease [22]. Indeed, activated and expanded B cells were detected in the thymus of MG patients [23,24], and clonally expanded B cells are found in the blood [25,26]. In EAMG, antibody-producing B cells expand in lymphoid organs, including the thymus, spleen and lymph nodes [27]. As such, we suspect that the B cell alterations of our model fit with the current model of MG. However, we also acknowledge that a systematic assessments of B cells (and other immune cell subsets) across lymphoid tissues in MG and EAMG is currently lacking.

In respect to the T cell compartment, in previous studies of EAMG, increased numbers of IL17 producing CD4 T cells were detected in the blood of EAMG mice [28]. In humans, CD4 T cells display increased production of IL4, IL17 and IL21 [29,30] and increased serum levels of these cytokines were detected [31]. In the context of EAMG, this dysregulated T cell response could create an inflammatory milieu conducive to the generation of antibody-producing B cells. IL4, for example, is known to promote B cell class switching to IgG, thereby enhancing antibody production [16,32]. IL17 can also indirectly support B cell activation and differentiation by stimulating stromal cells and also enhance isotype switching to IgG [16,33].

Interestingly, we also detected higher levels of CXCR5 on activated B cells in secondary lymphoid organs of EAMG mice. CXCR5 is a crucial signalling receptor required for the formation of lymphoid structures and the interaction of T and B cells [34]. In humans, thymic plasmablasts demonstrate increased expression of CXCR5 [35]. As such, CXCR5 acts as a chemotactic receptor recognizing chemokine C-X-C-ligand 13 (CXCL13) secreted from activated T cells enabling the migration of B cells into lymphoid organs [34,36]. These data implicate the CXCR5/CXCL13 signalling pathway as potential driver of B cell differentiation in EAMG, and potentially human MG. Indeed, a number of studies reported aberrant CXCR5/CXCL13 signalling in MG, particularly in cases associated with thymus pathology [37,38]. The importance of this pathway is underlined in EAMG and might be a therapeutic target of interest.

The contrasting roles of the spleen and lymph nodes could potentially underlie the differences observed in our EAMG model between these organs. While both organs are vital components of the immune system, their anatomical and functional distinctions lead to unique contributions to immune regulation. The spleen serves as a reservoir for immune cells and plays a crucial role in filtering blood, where it encounters circulating antigens [39]. In contrast, lymph nodes primarily facilitate immune cell interaction and antigen presentation, serving as hubs for immune surveillance and activation [40]. In respect to immune cells, the spleen serves as a major reservoir for B cells and monocytes, and functions as a site for the initiation of adaptive immune responses [39]. On the other hand, lymph nodes are crucial for the activation and proliferation of T cells. Following this line of thought, a pronounced immune response in the spleen might be for various reasons. The microenvironment within the spleen provides optimal conditions for B cell receptor engagement with antigens, leading to robust B cell responses [41]. Additionally, the spleen acts as a filtering organ, allowing for efficient trapping and concentration of antigens, which enhances B cell encounters with their cognate antigens [39,42]. Moreover, the spleen is rich in follicular dendritic cells and T helper cells, which provide crucial signals for B cell activation and antibody production. Taken together, these factors could contribute to a stronger B cell response in the spleen, including the engagement of CXCR5, in EAMG. However, further research is required to also assess the role of other immune cell subsets that our study lacked, e.g., regulatory B cells or innate immune cells.

A further limitation to this study is that previous models did not employ the same immune cell analysis and, as such, we cannot ascertain that the immunological profile of this model is reflective of previous EAMG models. Additionally, a limitation to this model is introduced by the exclusive use of recombinant AChR. While anti-AChR-antibody-mediated MG has gained considerable scientific interest, therapeutic options for MG induced by antibodies against the muscle-specific tyrosine kinase (MuSK) or the low-density lipoprotein 4 (LRP4) remain limited. In this model, we only assessed the effect of AChR, and cannot ascertain that recombinant MuSK or LRP4 produce viable models of EAMG. However, the reader is advised that previous studies in mice demonstrated that immunization with MuSK [43] or LRP4 [44] are feasible.

## 5. Conclusions

Conclusively, we report a robust model of EAMG employing a murine background and the use of recombinant AChR that might provide a platform for the further study of in-depth aspects of MG.

## Figures and Tables

**Table 1:** Flow cytometry antibodies.

Antigen	Fluorochrome	Clone	Supplier
CD4	BV421	RM4-5	Biolegend
CD8	BV510	53-6.7	Biolegend
CD3	BV650	17A2	Biolegend
CD185 (CXCR5)	BV421	L138D7	Biolegend
CD138	BV605	281-2	Biolegend
IgM	AF488	RMM-1	Biolegend
MHC II	PE/Cy5	M5/114.15.2	Biolegend
CD184 (CXCR4)	PE	QA16A08	Biolegend
CD19	PE Dazzle 594	6D5	Biolegend
CD25	PE/Cy7	PC61	Biolegend
CD23	APC	B3B4	Biolegend
IgD	AF700	11-26c.2a	Biolegend
CD45	APC/Cy7	30-F11	Biolegend
CD19	BV510	6D5	Biolegend
Interferon-γ	FITC	XM61.2	Biolegend
IL4	PE	11B11	Biolegend
PC7	CD25	PC61	Biolegend
IL17	APC	TC11-18H10.1	Biolegend
FoxP3	AF700	FJK-16s	Biolegend

## Data Availability

The datasets used and/or analysed during the current study are available from the corresponding author on reasonable request.
